# Extra-corporeal membrane oxygenation for refractory cardiogenic shock after adult cardiac surgery: a systematic review and meta-analysis

**DOI:** 10.1186/s13019-017-0618-0

**Published:** 2017-07-17

**Authors:** Maziar Khorsandi, Scott Dougherty, Omar Bouamra, Vasudev Pai, Philip Curry, Steven Tsui, Stephen Clark, Stephen Westaby, Nawwar Al-Attar, Vipin Zamvar

**Affiliations:** 1 Department of Cardiac Surgery and Transplantation, Golden Jubilee National Hospital, Glasgow, UK; 2 Department of Cardiology, Ninewells hospital, Dundee, UK; 3 Medical Statistics, Trauma, Audit & Research Network, University of Manchester, Salford Royal NHS foundation trust, Manchester, UK; 4 Department of Cardiovascular and Thoracic Surgery, Kasturba Medical College, Manipal University, Manipal, India; 5 Department of Cardiac Surgery and Transplantation, Papworth hospital, Cambridge, UK; 6 Department of Cardiac surgery and Transplantation, Freeman hospital, Newcastle, UK; 7 Department of Cardiac Surgery, Oxford Heart Center, John Radcliffe Hospital, Oxford, UK; 8 Department of Cardiothoracic Surgery, Royal Infirmary of Edinburgh, Edinburgh, UK

**Keywords:** Extra-corporeal membrane oxygenation, Postcardiotomy, Cardiogenic shock

## Abstract

**Background:**

Postcardiotomy cardiogenic shock (PCCS) refractory to inotropic support and intra-aortic balloon pump (IABP) occurs rarely but is almost universally fatal without mechanical circulatory support. In this systematic review and meta-analysis we looked at the evidence behind the use of veno-arterial extra-corporeal membrane oxygenation (VA ECMO) in refractory PCCS from a patient survival rate and determinants of outcome viewpoint.

**Methods:**

A systematic review was performed in January 2017 using PubMed (with no defined time period) using the keywords “postcardiotomy”, “cardiogenic shock”, “extracorporeal membrane oxygenation” and “cardiac surgery”. We excluded papers pertaining to ECMO following paediatric cardiac surgery, medical causes of cardiogenic shock, as well as case reports, review articles, expert opinions, and letters to the editor. Once the studies were collated, a meta-analysis was performed on the proportion of survivors in those papers that met the inclusion criteria. Meta-regression was performed for the most commonly reported adverse prognostic indicators (API).

**Results:**

We identified 24 studies and a cumulative pool of 1926 patients from 1992 to 2016. We tabulated the demographic data, including the strengths and weaknesses for each of the studies, outcomes of VA ECMO for refractory PCCS, complications, and APIs. All the studies were retrospective cohort studies. Meta-analysis of the moderately heterogeneous data (95% CI 0.29 to 0.34, *p* < 0.01, *I* ^*2*^ = 60%) revealed overall survival rate to hospital discharge of 30.8%. Some of the commonly reported APIs were advanced age (>70 years, 95% CI −0.057 to 0.001, *P* = 0.058), and long ECMO support (95% CI −0.068 to 0.166, *P* = 0.412). Postoperative renal failure, high EuroSCORE (>20%), diabetes mellitus, obesity, rising lactate whilst on ECMO, gastrointestinal complications had also been reported.

**Conclusion:**

Haemodynamic support with VA ECMO provides a survival benefit with reasonable intermediate and long-term outcomes. Many studies had reported advanced age, renal failure and prolonged VA ECMO support as the most likely APIs for VA ECMO in PCCS. EuroSCORE can be utilized to anticipate the need for prophylactic perioperative VA ECMO in the high-risk category. APIs can be used to aid decision-making regarding both the institution and weaning of ECMO for refractory PCCS.

## Background

Postcardiotomy cardiogenic shock (PCCS) refractory to inotropic support and intra-aortic balloon counter pulsation (IABP) is an infrequent but almost universally fatal condition without mechanical circulatory support (MCS) [1–5]. Veno-Arterial (VA) extra-corporeal membrane oxygenation (ECMO) has been utilized as a salvage MCS after cardiac surgery for almost 50 years [6, 7]. The decisions surrounding when best to institute or withdraw such invasive and resource-intensive therapy remains controversial and there are no universally agreed upon guidelines on the indications for this therapy. VA ECMO in the context of refractory PCCS is mainly instituted as a temporizing measure as a *“bridge to recovery”* [5, 8, 9]. However, it has also been utilized as a *“bridge to decision”* and *“destination therapy”* with long-term implantable devices (e.g. left ventricular assist device, LVAD), and more rarely in the UK, “bridge to orthotopic heart transplantation (OHT)” [8–10]. Nevertheless, ECMO carries with it a significant morbidity rate, often associated with prolonged hospital stays and poor quality of life for the survivors after hospital discharge [5, 11].

In this systematic review and meta-analysis we have looked at the survival rate following VA ECMO for intractable PCCS in adults and some of the most commonly and consistently reported adverse prognostic indicators (API) in this group of patients.

## Methods

A comprehensive literature search was performed in January 2017 using OVID/MEDLINE (PubMed) for all articles published in the English language in peer-reviewed journals. The inclusion criterion was post-cardiotomy VA ECMO in adults. We excluded all articles pertaining to VA ECMO for paediatric cardiac surgery as well as non-surgical indications for the use of this therapeutic modality e.g. myocarditis or cardiomyopathy. We also excluded case reports, review articles, expert opinions, and letters to the editor.

The search was performed, with no limit to the year of publication, using the following three search strategies:

Search 1: “*(((Extracorporeal membrane oxygenation) AND thoracic surgery) AND cardiac surgical procedures) AND shock, cardiogenic*”,

Search 2: “*((Extracorporeal membrane[Title]) OR ECMO[Title]) AND cardiac surgery[Title/Abstract]”.*

Search 3: **“** *(((Extracorporeal membrane[Title]) OR ECMO[Title]) AND postcardiotomy[Title/Abstract])”.*

The first search strategy yielded 179 articles, the second search strategy yielded 149 articles and the third search strategy yielded 103 articles. The authors assessed the abstracts in all three searches. The “*preferred reporting items for systematic reviews and meta-analyses*” (“PRISMA”) guideline [12] was followed. When it was not possible to ascertain article suitability from its abstract alone we obtained the full text article for further assessment.

As this was a systematic review, ethical board review was not required. The primary outcome measure was determining the survival benefit following the institution of VA ECMO for refractory PCCS. The secondary outcome measure was identifying the most commonly reported APIs. The demographic data, APIs that had reached statistical significance (*P* < 0.05) in each study and crude data on outcome and follow-up, where available, were collated from each study. We then performed meta-regression to assess whether the APIs identified were significantly associated with survival.

## Results

We identified 24 retrospective cohort studies that met the inclusion criteria from 1992 to 2016 in the English language literature. These studies as well as their strengths and weaknesses, are summarized in Table 1. In this review a cumulative total of 1926 patients required VA ECMO for PCCS.

**Table 1:** Summary of outcomes of studies examining patients with PCCS treated with VA-ECMO

Author (year published) & country	Historical period	Number of patients & Indications for surgery	Outcomes with key results	Comments
Khorsandi et al. [29] (2015), United Kingdom	4/1995–4/2015	15 patients -AVR (*n* = 3) -CABG (*n* = 3) -CABG & AVR (*n* = 1) -Aortic dissection/transection (*n* = 3) -MV repair/MVR & CABG (*n* = 2) -Miscellaneous (*n* = 3)	Age range: 34–83 years (median 71) 30-day survival: 37.5% Survival to hospital discharge: 31.25% 24-month survival: 31.25 Functional status: NYHA status I-II	Authors reported acceptable functional outcome post VA-ECMO Weaknesses: limited sample size; retrospective study
Doll et al. [13] (2004), Germany	11/1997–7/2002	219 patients -CABG (*n* = 119) -CABG & AVR (*n* = 240) -MVR (*n* = 110 -Other (*n* = 44)	Average age: 61.3 years +/− 12.1 Mean period on ECMO: 2.8 +/− 2.2 days Weaned from ECMO: 61% (*n* = 133) 30-day mortality: 76% (*n* = 167) Discharged from hospital after 29 +/− 24 days: 39% (*n* = 52) 5-year follow-up: 74% (*n* = 37) were alive	Authors concluded that VA-ECMO is an acceptable life-saving measure in high-risk patients with refractory PCCS Weaknesses: limited study size; retrospective study
Khorsandi et al. [20] (2016), United Kingdom	4/1995–4/2015	23 patients -AVR (*n* = 8) -CABG (*n* = 6) -CABG & AVR (*n* = 2) -Aortic dissection/transection (*n* = 4) -MVR/MV repair & CABG (*n* = 3)	Age range: 34–83 years (median 51) 30-day survival: 39% (*n* = 9) Survival to hospital discharge: 35% (*n* = 8) 12-month follow up: 100% survival Functional status: NYHA I-II	Authors concluded that VA-ECMO has a high rate of systemic and device-related complications with a definite survival benefit
Rastan et al. [5] (2010), Germany	5/1996–5/2008	516 patients: -CABG (37.4%) -CABG & AVR (16.6%) -AVR (14.3%) -Heart/lung transplant (6.5%) -Other (25%)	Age range: 18–84 years (mean 63.5) Mean ECMO duration: 3.28 days +/− 2.85 Weaned from ECMO: 63.3% Discharged home: 24.8% Complications: 65% renal complications; 58% major bleeding requiring re-intervention; 17.4% stroke; 18% GI complications	Authors concluded that VA-ECMO is an acceptable treatment for refractory PCCS in those patients that would otherwise die Weaknesses: limited sample size; retrospective study
Slottosch et al. [21] (2013), Germany	2006–2010	77 patients: -CABG (*n* = 43) -Valve (*n* = 10) -CABG & Valve (*n* = 11) -Aortic surgery (*n* = 5) -Heart transplant (*n* = 2) -Other (*n* = 6)	Mean age: 60 years +/− 13 Weaned from ECMO: 62% 30-day mortality: 70% Predictors of mortality: advanced age (*p* = 0.003); rising serum lactate, prolonged ECMO course and ECMO-GI complications were independent predictors of 30-day mortality (*p* < 0.05) Complications of ECMO: limb ischaemia (20.8%), renal failure (68.8%), & reopening for bleeding (29.9%)	High-quality study No long-term follow-up outcomes included Needs long-term quality of life assessment
Hsu et al. [38] (2010), Taiwan	1/2001–12/2006	51 patients: -CABG (*n* = 27) -Valve surgery (*n* = 11) -CABG & valve (*n* = 7) -Heart transplant (*n* = 4) -Other (*n* = 2)	Average age: 63 years +/− 15.7 Mean duration of ECMO: 7.5 days +/− 6.7 30-day mortality: 49% 3-month mortality: 65% 1-year mortality: 71%	Authors concluded that VA-ECMO provides good support post PCCS Study weaknesses: small sample size; longer-term follow-up required; quality of life assessment not included
Ko et al. [19] (2002), Taiwan	8/1994–5/2000	76 patients: -CABG (*n* = 37) -CABG a & valve (*n* = 6) -Isolated valve (*n* = 14) -Heart transplant (*n* = 12) -Congenital surgery (*n* = 3) -LVAD (*N* = 2) -Aortic surgery (*n* = 2)	Mean age: 56.8 years +/− 15.9 Weaned from ECMO: 60.5% (*n* = 46) Survival to hospital discharge: 26.3% (*n* = 20) Functional status: all survivors NYHA I-II on follow-up period of 33+/−22 months	Strengths: good intermediate-term follow-up Weaknesses: small sample size
Muehrcke et al. [3] (1996), USA	9/1992–7/1994	22 patients: -CABG (*n* = 8) -Valve (*n* = 6) -Heart transplant (*n* = 4) -Post-infarction VSD (*n* = 2) -Miscellaneous (*n* = 2)	Average age: 47.3 years +/− 16.4 (range 5–72) Weaned from ECMO: 30.4% (*n* = 7) Subsequent heart transplant: 13.6% (*n* = 3) Complications: major haemorrhage, leg ischaemia, renal failure, thrombus formation, and stroke	Weaknesses: limited sample size, no long-term follow-up data
Santarpino et al. [14] (2015), multicenter European study	2005–2015	20 patients, from 11 European centers -CABG (*n* = 85)	Average age: 64.6 years +/− 10.3 Survival to hospital discharge: 40% (*n* = 8) 1-year survival: 29.3% Complications: stroke (40%), reopening for bleeding (60%), dialysis for renal failure (35%), DSWI (30%)	Salvage CABG has high rate of immediate mortality ECMO for refractory PCCS has encouraging results
Saeed et al. [39] (2015), Germany	1/2013–7/2014	9 patients: -CABG & valve replacement (*n* = 5) -CABG (*n* = 4)	Average age: 65 years +/− 14 Weaned from ECMO: 44% (*n* = 4) Survival to hospital discharge: 22% (*n* = 2) Complications: renal failure (89%, *n* = 8)	Weaknesses: small sample size
Sajjad et al. [40] (2012), Saudi Arabia	1/2007–12/2009	19 patients: - Emergency (*n* = 11) -Urgently (*n* = 5) -Electively (*n* = 3)	Age range: 21–79 years (mean 55.6) Unable to wean (died on ECMO): 63% (*n* = 12) 30-day mortality: 94.7% Survival to hospital discharge: 5.3%	Authors concluded that ECMO is costly, prolongs ICU stay and delays imminent death in most patients
Mikus et al. [22] (2013), Italy	2007–2014	14 patients: -AVR and/or MVR (*n* = 6) -CABG (*n* = 6) -Bentall procedure (*n* = 3)	Mean age: 53.1 years +/− 14.3 (range 25–70) Successful weaning: 50% (*n* = 7) Survival to hospital discharge: 42.8% (*n* = 6) Complications: mediastinal bleeding (64.3%), renal failure (50%), sepsis (42.8%), pneumonia (28.6%)	Authors concluded that VA-ECMO with Levitronix CentriMag^R^ is a reliable and easy to apply life-saving mechanical support which can be applied to bridge postcardiotomy patients to decision
Unosawa et al. [27] (2013), Japan	04/1992–06/2007	47 patients: -CABG (*n* = 19) -Valve (*n* = 8) -CABG & Valve (*n* = 2) -Aortic surgery (*n* = 5) -Valve & aortic surgery (*n* = 1) -Aortic surgery & CABG (*n* = 3) -Aortic root replacement (*n* = 2) -Post-infarction VSD (*n* = 5) Pulmonary embolectomy (*n* = 2)	Average age: 64.4 years +/− 12.5 (range 22–83) Weaned from ECMO: 60.7% (*n* = 29) Survival to hospital discharge: 48% (*n* = 14) 30-day survival: 34% 1-year survival: 29.8% 10-year survival: 17.6% Independent risk factors for mortality: incomplete sternal closure (*p* = 0.049) and ECMO duration >48 h (*p* = 0.027)	Authors concluded that VA-ECMO for refractory PCCS is associated with high morbidly and mortality but that survivors have acceptable long-term survival Strengths: long follow-up period
Pokersnik et al. [41] (2012), USA	01/2005–12/2010	49 patients. Group 1 (*n* = 11): Biomedicus pump with an affinity oxygenator Group 2 (*n* = 11): Biomedicus pump with a Quadrox D oxygenator Group 3 (*n* = 27): Rotaflow pump with a Quadrox D oxygenator	Average age: 65 years +/− 13 Weaned from ECMO: -Group 1: 63.6% -Group 2: 45.5% -Group 3: 55.6% In-hospital survival: -Group 1: 27.3% -Group 2: 27.3% -Group 3: 33.3%	Authors concluded that outcomes for patients undergoing ECMO for PCCS remain poor in all categories
Moreno et al. [42] (2011), Spain	11/2006–12/2009	12 patients -Cardiac surgery (*n* = 8) -Heart transplant (*n* = 4)	Mean age: 56.8 years (standard deviation 9.1) Mean duration on ECMO: 5.4 days Survival to hospital discharge: 50%	Authors concluded that VA-ECMO provided viable temporary circulatory support
Wu et al. [17] (2010), Taiwan	2003–2009	110 patients: -CABG (*n* = 31) -Valve (*n* = 16) -Multiple valves (*n* = 26) -Combined valve and other (*n* = 19) -Aortic surgery (*n* = 8) -Post-infarction VSD (*n* = 3) -Pulmonary endarterectomy (*n* = 4) OHT (*n* = 3)	Average age: 60 years +/− 14 Weaned from ECMO: 61% (*n* = 67) Survival to hospital discharge: 42% (*n* = 46) Adverse prognostic indicators: age > 60 years, renal failure, serum bilirubin >6 mg/dL, and duration of ECMO >110 h; persistent heart failure (EF <60%) was a predictor of mortality after hospital discharge	Authors concluded that VA-ECMO has a definite survival benefit Strengths: adverse prognostic indicators were reported
Elsharkawy et al. [16] (2010), USA	1/1995–12/2005	233 patients: -CABG (*n* = 86) -Any valve (*n* = 69) -AVR/repair (*n* = 42) -MV repair/MVR (*n* = 44) -TV repair/TVR (*n* = 16)	Survivors’ IQR: 45.1–61.4 (median 53.5) Non-survivors’ IQR: 52.1–66.3 (median 59.7) Survival to hospital discharge: 36% Associated with higher mortality rate: older age, known diabetes, CABG, longer CPB time Associated with reduced hospital morality: younger age	Authors concluded that patient selection for salvage VA-ECMO for refractory PCCS remains difficult as the variables identified in the study are not easily modifiable and do not appear to be “robust”
Bakhtiary et al. [18] (2008), Germany	1/2003–11/2006	45 patients: -CABG (*n* = 20) -LVAD (*n* = 5) -OHT (*n* = 1) -CABG & Post-infarction VSD (*n* = 3) -CABG & MV repair (*n* = 5) -AVR (*n* = 2) -CABG & AVR (*n* = 3) -Miscellaneous (*n* = 5)	Average age: 60.1 years +/− 13.6 Weaned from ECMO: 55% (*n* = 25) 30-day mortality: 55% (*n* = 25) In-hospital morality: 71% (*n* = 32) Survival to hospital discharge: 29% (*n* = 13) 3-year survival: 77% (*n* = 10) with NYHA class II (*n* = 6), NYHA class IV (*n* = 4) Improved survival: absence of pulmonary hypertension and use of IABP (*p* = 0.04)	Authors concluded that VA-ECMO provides sufficient cardiopulmonary support. Peripheral cannulation techniques and reduced anticoagulation could reduce bleeding rates
Doll et al. [8] (2003), Germany	11/1997–02/2000	95 patients: -CABG (*n* = 63) -AVR (*n* = 16) -CABG & AVR (*n* = 8) -Others (*n* = 8)	Average age: 59.8 years +/− 13.3 Weaned from ECMO: 47% (*n* = 45) Survival to hospital discharge: 29% (*n* = 28) Mortality rates for CABG & AVR on ECMO: 100% (*p* < 0.05) Complications: renal failure (64%), re-exploration for haemorrhage (62%), & limb ischaemia (16%)	Authors concluded that “short term” ECMO support is a suitable technique for short-term low cardiac out states
Wang et al. [43] (1996), Taiwan	10/1994–10/1995	18 patients: -CABG (*N* = 7) -CABG & Valve (*n* = 3) -OHT (*n* = 3) -Valve (*n* = 2) -Miscellaneous (*n* = 3)	Average age: 46.5 years +/− 24.6 Weaned from ECMO: 52.6% (*n* = 10) Survival to hospital discharge: 33% (*n* = 6) in “good condition” Complications: leg ischaemia (*n* = 3), bleeding (*n* = 4), renal failure (*n* = 3), and tube rupture (*n* = 1)	One patient received 2 runs of ECMO This cohort included routine adult cardiac surgery as well as heart transplants
Magovern et al. [44] (1994), USA	10/1991–10/1993	21 patients Divided into 3 categories: -Cat 1: after CABG (*n* = 14) -Cat 2: MV surgery (*n* = 3) -Cat 3: after open heart surgery & prolonged CPR (*n* = 4)	Mean age: 61.6 years +/− 2.2 (33–78) Survival to hospital discharge: -Cat 1: 80% (0% for both categories 2 & 3) -Total survival to hospital discharge: 52% Complications: stroke, renal failure, and mediastinitis	Authors commented that VA-ECMO in the context of MV surgery does not decompress the LV (where there is often concurrent LV distension), thus is not effective
Saxena et al. [45] (2015), USA	2003–2013	45 patients Additional inclusion criteria: age > 70 years: -Valve repair/replacement (*n* = 16) -Valve & CABG (*n* = 13) -Other (*n* = 16)	Mean age: 76.8 years +/− 4.6 Mortality whilst on ECMO: 46.6% (*n* = 21) Weaned from ECMO: 53.3% (*n* = 24) Survival to hospital discharge: 24% (*n* = 11) Complications: renal failure 44.4% (*n* = 30), pneumonia 26.7% [12], & sepsis 24.4% (*n* = 11) Adverse prognostic indicators: preoperative AF, CKD, lactic acidosis on ECMO, persistent coagulopathy	Total 47 runs of ECMO (two patients each received two runs) Authors concluded that VA-ECMO for PCCS confers high morbidity & mortality rates. However, it provides a last line of support for patients that would otherwise die
Li et al. [15] (2015), China	01/2011–12/2012	123 patients: -CABG (*n* = 44) -CABG & other (*n* = 15) -Valve (*n* = 40) -OHT (*n* = 11) -Other (*n* = 13)	Mean age: 56.2 years +/− 11.8 (range 18–76) Weaned from ECMO: 56% Survival to hospital discharge: 34.1%	Predictors of in-hospital mortality: advanced age, female sex, elevated mean lactate and lactate clearance (*p* < 0.05)
Yan et al. [46] (2010), China	2004–2008	67 patients: -CABG +/− Valve (*n* = 49) -OHT (*n* = 9) -Adult CHD (*n* = 5) -Other (*n* = 5)	Average age: 50.5 years +/− 13.6 Survival to hospital discharge: 49% Prognostic indicators: mortality was much higher amongst patients who received RRT than those that did not (73% vs 32%, *p* = 0.001)	Authors concluded that renal failure is a major ECMO-related complication after PPCS and is associated with a significant mortality rate

*Abbreviations*: *ECMO* Extra-corporeal membrane oxygenator, *CABG* coronary artery bypass grafting, *IABP* Intra-aortic balloon pump, *LVAD* Left ventricular assist device, *RVAD* Right ventricular assist device, *BiVAD* Biventricular assist device, *NYHA* New York Heart Association, *MODS* Multi-organ dysfunction syndrome, *VA* veno-arterial, *GI* gastrointestinal, *AVR* aortic valve replacement, *MVR* Mitral valve replacement, *CI* cardiac index, *CPB* Cardiopulmonary bypass, *AS* aortic stenosis, *MI* Myocardial infarction, *LMS* left main stem coronary artery, *PVD* peripheral vascular disease

*PCCS* Post cardiotomy cardiogenic shock, *ICU* intensive care unit, *LV* left ventricle, *RV* right ventricle, *RRT* renal replacement therapy, *Pts* patients, *OHT* orthotopic heart transplantation, *CHD* congenital heart disease, *MV* mitral valve, *MVR* mitral valve replacement, *TV* tricuspid valve, *TVR* tricuspid valve replacement

### Survival and follow-up

In the largest cohort with the longest follow up available in the literature, Rastan et al. reported results from 516 patients undergoing salvage ECMO for PCCS over a 12-year period from 1996 to 2008. They reported a survival to hospital discharge rate of 24.8% and a 13.7% 5-year survival [5]. In another large study Doll et al. reported results from 219 patients that underwent VA ECMO for PCCS from 1997 to 2002. They reported a 39% survival to hospital discharge and 17% 5-year survival [13].

In a multinational European study, Santarpino et al. collated data from 11 European cardiac surgical centers with cumulative results from 85 adult patients. Survival rate to hospital discharge was reported as 40% with a 1-year survival rate of 29.3% [14].

Li et al. reported on 123 adult patients who were salvaged with VA ECMO for refractory PCCS. They noted that 56% of patients were successfully weaned from VA ECMO and 34.1% survived to hospital discharge [15]. Elsharkawy et al. reported results from 233 patients with survival to hospital discharge was reported at 36% [16]. In another cohort study Wu et al. reported outcomes from 110 patients of whom 61% were successfully weaned and 42% were successfully discharged home [17].

Bakhtiary et al. reported a 55% survival to ECMO decannulation in their 45 patient cohort study, with a total in-hospital mortality rate of 71% for the cohort. In 3 years, 77% of the survivors were still alive [18]. Ko et al., analyzed the outcomes of 76 patients who underwent ECMO for intractable PCCS. Although 60.5% of patients survived to ECMO decannulation, 26.5% survived to hospital discharge. On 32+/−22 months follow-up, all survivors were of NYHA I-II functional status [19]. In a smaller cohort conducted by some of the authors of this systematic review, 35% survival to hospital discharge was observed and all survivors were still alive at 12 months with NYHA class I-II [20].

### Complications

Major haemorrhage was the most commonly reported complication after institution of VA ECMO. It led to re-intervention in almost half of the patients in a few good quality studies [3, 5, 13, 14, 19, 20]. Factors such as the insult of the original operation, heparin infusion and heparin coated circuits are known to be the primary causes of bleeding [13]. Renal failure requiring renal replacement therapy (RRT) was the next most commonly reported complication [3, 5, 13, 20–22]. Stroke and sepsis also followed amongst the commonly encountered complications (see Table 1). Distal limb ischaemia is one of the most dreaded complications because if often leads to major morbidity. The largest study in this systematic review reported that almost 20% of patients with peripheral VA ECMO developed a degree of distal limb ischaemia. However, the use of a polyethylene terephthalate tube graft (e.g. Dacron^R^) as a sewn side arm to the femoral or axillary arteries or the distal leg perfusion cannula reduced distal limb ischaemia and fasciotomy for compartment syndrome rate by almost 40% [5].

### Adverse prognostic indicators

APIs that reached statistical significance and were directly associated with mortality in each study are summarized in Table 1. Advanced age (typically >70 years), although perhaps not in isolation [5, 15, 17, 21, 23], was the most commonly reported API. Development of renal failure, whilst on VA ECMO that required RRT had a strong association with mortality [5, 16, 23–25]. While this common complication in the context of VA ECMO for refractory PCCS is usually multifactorial. It is imperative to determine the most likely underlying aetiology/ies for renal dysfunction as early as possible. The following potential aetiologies should be identified and corrected as early as possible postoperatively: renal hypoperfusion due to poor forward flows whilst on VA ECMO, acute tubular necrosis due to prolonged hypotension preceding institution of support or syndrome of inappropriate anti-diuretic hormone secretion (SIADH) [26]. It is worth noting that the use of loop diuretics to address fluid overload or poor urine output leads to worsening renal dysfunction [26]. Rising serum lactate whilst on VA ECMO [5, 15, 21] has also been reported as a strong predictor of mortality. One study [5] advocated use of sodium bicarbonate infusion at an early stage to reduce metabolic acidosis and the organ damage that might ensue. Diabetes mellitus [5, 23], obesity [5, 23], gastrointestinal complications whilst on ECMO, high EuroSCORE (>20) and protracted ECMO support (>48 h) were also amongst the commonly reported APIs [17, 21, 26, 27]. It must however be acknowledged that prolonged ECMO support might reflect the complexity of the original operation and the patient’s poor clinical.

Although EuroSCORE was widely reported in the studies, it was quoted in its three versions (i.e. the additive EuroSCORE, logistic EuroSCORE and EuroSCORE II) over nearly 3 decades covered by this systematic review. This variation made meta-analysis for EuroSCORE as an API not possible.

### Meta-analysis of survival to hospital discharge

**Fig. 1:**
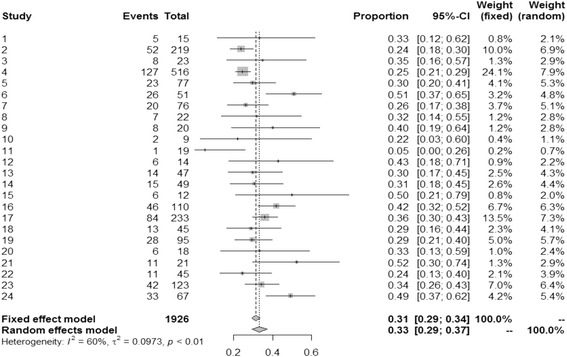
Demonstrates the forest plot of the studies and the variables included in Table 2, describing each studies proportion of survivors (CI 95%)

We found that of the pooled total of 1926 patients, 594 (30.8%) survived to hospital discharge. We performed a meta-analysis on the outcome of interest i.e. the proportion of survivors. The software R was used with the package “metaprop”**. Meta-analysis is conducted to estimate the true unknown success rate of a procedure, by combining the results of several studies. Table 2 summaries the variables included in the meta-analyses. Figure 1 shows the forest plot describing each of the study’s proportion of survivors with their 95% confidence interval (CI).

**Table 2:** Summary of the information utilized to perform the meta-analysis

Study ID	Author	Country	Year	Average age (yrs)	Preoperative IABP usage (no. pts)	Renal Failure (no. pts)	Mean ECMO support (days)	Diabetes mellitus (no. pts)	Survivors	Patient no.
1	Khorsandi	UK	2015	71	15	2	5.5	0	5	15
2	Doll	Germany	2004	61.3	144	122	NA	68	52	219
3	Khorsandi	UK	2016	51	23	7	5.5	NA	8	23
4	Rastan	Germany	2010	63.5	383	81	4.6	168	127	516
5	Slottosch	Germany	2013	60	72	26	3.3	18	23	77
6	Hsu	Taiwan	2010	63	51	16	7.5	19	26	51
7	Ko	Taiwan	2002	56.8	44	28	NA	NA	20	76
8	Muehrcke	USA	1996	47.3	17	12	2.5	NA	7	22
9	Santarpino	Europe	2015	64.6	16	7	4.5	25	8	20
10	Saeed	Germany	2015	65	9	8	8.5	NA	2	9
11	Sajjad	UK	2012	55.6	19	NA	4	NA	1	19
12	Mikus	Italy	2013	53.1	14	7	5	4	6	14
13	Unosawa	Japan	2013	64.4	39	15	2.6	9	14	47
14	Pokersnik	USA	2012	65	29	16	4	19	15	49
15	Moreno	Spain	2011	56.8	12	4	5.4	5	6	12
16	Wu	Taiwan	2010	60	110	46	6	NA	46	110
17	Elsharkawy	USA	2010	53.5	22	101	NA	50	84	233
18	Bakhtiary	Germany	2008	60.1	30	39	6.4	38	13	45
19	Doll	Germany	2003	59.8	95	64	2.8	NA	28	95
20	Wang	Taiwan	1996	46.5	9	3	5.1	NA	6	18
21	Magovern	USA	1994	61.6	21	4	1.8	4	11	21
22	Saxena	USA	2015	76.8	45	30	4.2	17	11	45
23	Li	China	2015	56.2	73	29	4.4	NA	42	123
24	Yan	China	2010	50.5	18	30	3.1	NA	33	67

*Abbreviations*: *NA* not available, *pts* patients, *IABP* intra-aortic balloon pump, *ECMO* extra-corporeal membrane oxygenation, *no*. numbers, *yrs.* years

**R Core Team (2013). R: A language and environment for statistical computing. R Foundation for Statistical Computing, Vienna, Austria. URL http://www.R-project.org/

Heterogeneity is present as defined by a statistically significant *I* ^*2*^ = 60%, which represents the percentage of variability in the effect estimates due heterogeneity rather than random error. A value of *I* ^*2*^ between 30 and 60% is considered as a moderate heterogeneity. By looking at the forest plot, one can observe that study 11 is well out from the rest. By removing study 11, the *I* ^*2*^ value dropped to 59%. The overall proportion of survivors is 31% (95% CI 0.29 to 0.34, *p* < 0.01, *I* ^*2*^ = 60%), but when considering random effect it raises to 33%.

### Meta-regression of the adverse prognostic indicators

Meta-regression on the effects of moderators such as age, usage of pre-VA ECMO IABP support and duration of VA ECMO support was performed as these were the most commonly and consistently reported variables by most of the studies. Analysis showed that heterogeneity had been observed due to random sampling and variation in the methods used for the studies (Table 3). In order to account for at least part of the heterogeneity, mixed effects models are used by including moderators. This was also carried out using the package “metafor” in R software. By including the moderators the heterogeneity statistic *I* ^*2*^, which represents presence of variation in an inter-study effect size (proportion of survivors) dropped to 52.2% from the model without moderators (previously 60% see forest plot, Fig. 1), which is classified as moderate. We found that none of the coefficients of the moderators are statistically significant (Table 3), which means that there is no moderating effect of the mean age, usage of pre-VA ECMO IABP and the mean number of days on ECMO on the effect size. One of the reasons of the lack of power for the test on the coefficients is the small sample sizes used in the studies.

**Table 3:** Shows the coefficients of the meta-regression for each of the moderators in a logit scale

Covariate	Coefficient	Standard	95%	95%	Z-value	2-sided
	(logit scale)	Error	Lower	Upper		*P*-value
Intercept	0.669	0.824	−0.946	2.284	0.810	0.417
Mean Age (yr)	−0.028	0.015	−0.057	0.001	−1.900	0.058
IABP rate	0.206	0.446	−0.667	1.080	0.460	0.643
Mean ECMO (days)	0.049	0.060	−0.068	0.166	0.820	0.412

**Fig. 2:**
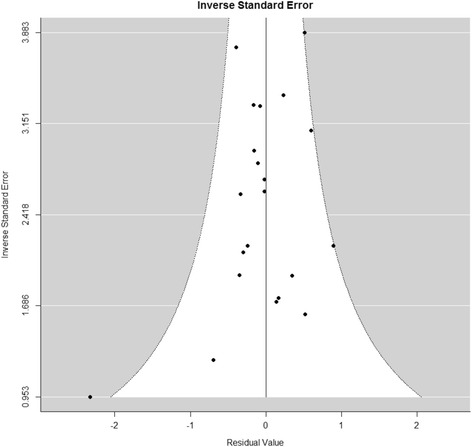
Demonstrates the funnel plot on the analysis on publication bias, which shows no departure from symmetry, hence absence of bias (Egger’s test *p* = 0.556)

Furthermore, we looked at the possibility of publication bias in the meta-analysis. Our analysis showed no departure from symmetry in the funnel plot (Fig. 2), hence absence of bias. This claim is supported by the Egger’s test (*p* = 0.556).

## Discussion

Our systematic review is the first of its kind to be published, analyzing the efficacy of VA ECMO as a salvage modality for refractory PCCS.

Refractory PCCS typically transpires at the end of a complex and prolonged operation [28]. It can also occur in an otherwise routine operation [5, 29] that has encountered an unexpected technical difficulty e.g. an iatrogenic injury to a vital structure during the course of an operation. In either case the patient cannot be weaned from cardiopulmonary bypass (CPB) with survivable haemodynamic and arterial blood gas parameters, despite multiple inotropic support agents and IABP [30]. Furthermore, adding to the predicament, such cases typically encroach into “out-of-hours” [31] when the operating theatre team is fatigued and there is a relative lack of availability of technical assistance and experienced advice.

### Why VA ECMO?

VA ECMO for refractory PCCS is typically established centrally i.e. arterial line through the ascending aorta and the venous line through the right atrium as part of the continuum with CPB at the end of the operation [5, 29]. Although in principal the conduct of CPB and VA ECMO are similar, there are several important advantages in using VA ECMO in the context of refractory PCCS [32]. The CPB achieves full VA bypass at lower flow rates (2–2.4 L/min/m^2^) with haematocrit levels of around 20%, leading to lower than physiological systemic oxygen delivery (DO_2_) [32]. A large dose (300–400 Units/kg) of unfractionated heparin (UH) is required to run CPB, as it is an “open circuit” with a venous reservoir (stagnant blood). In addition, full VA bypass is said to cause stasis of blood in the cardiac chambers and the pulmonary circulation warranting higher activated clotting times (ACT). VA ECMO on the other hand is more physiological. It uses partial VA bypass through a “closed circuit” (without a venous reservoir) and shorter tubing, typically in normothermia, with normal haematocrit levels, aiming for near normal DO_2_. As VA ECMO allows venous return to the heart, it allows cardiac ejection and less risk of thrombosis, thereby requiring minimal doses of UH as compared to CPB. This in-turn leads to fewer rates of postoperative bleeding complications requiring re-exploration whilst on MCS. VA ECMO is more versatile and is more easily manageable in the intensive care unit (ICU) setting for often-prolonged periods of time (i.e. days-weeks), unlike CPB, which is geared more towards short-term support (i.e. a few hours) [32]. The VA ECMO line tubing can be tunneled through the skin to allow chest closure. This maybe significant in that “incomplete sternal closure” has been reported as an independent predictor of mortality, as identified in a study by Unosawa et al. [27]. However, line change over to other sites, with axillary arterial, femoral arterial and femoral venous cannulation sites have previously been reported [5, 13]. Occasionally combined right atrial and femoral venous cannulation have been used to improve venous drainage [5]. Vascular access can be established with either surgical cut down or by Seldinger techniques. Some sew a side arm tube graft to the artery or use a distal perfusion cannula to reduce the risk of distal limb ischaemia and compartment syndrome (see complications) [5, 13].

Although ECMO is a valuable salvage modality, it is expensive [33]. It is very resource intensive due to its high demand on the ICU staff. It requires skilled staff performing high frequency monitoring for its safe application and maintenance [33]. These factors can pose a significant burden particularly on small and intermediate sized cardiac surgical centers, where the work force and bed capacity are both limited [30]. ECMO patients, on average, require more prolonged ICU stay than elective cases thereby leading to increased cancellation of elective and sometimes urgent cardiac surgical operations. As such, indiscriminant use of VA ECMO can result in major disturbance to an already stretched service [30].

Some centers in the UK rely on transplant centers for advice regarding whether or not to place PCCS patients on VA ECMO. If these patients are commenced on ECMO, they are transferred over to the larger transplant centers for further management [30, 34]. Critics had argued that, if only transplant units are funded to provide ECMO for PCCS support, similar patients at non-transplant cardiac surgical centers are denied of a life saving therapy [35]. In reality the use of VA ECMO for PCCS is not formally commissioned by the National Health Service (NHS) in the UK and the cost of treatment has to be absorbed by individual hospitals.

Kashani et al. [36], published an abstract on a systematic review covering only 11 case series with a cumulative pool of 1328 patients whereas our review covers 24 studies with a cumulative pool of 1926 patients. The survival rate to hospital discharge reported by Kashani et al. of 31.48% was comparable to our report of 30.8%. In their systematic review, they found similar APIs to our systematic review such as advanced age, elevated serum lactate after initiation of ECMO and renal failure [36]. Our meta-regression of APIs such as mean age, pre-VA ECMO use of IABP, effect of renal failure and mean ECMO duration however showed no statistically significant correlation between these parameters and survival, mainly due to the small sample sizes and presence of wide heterogeneity amongst study populations (Table 3).

This topic remains a controversial one with ethical and financial considerations for any cardiac surgical service. The decision as to whether or not to institute VA ECMO in the setting of PCCS remains difficult. Institution of VA ECMO for PCCS is usually not planned and the aetiology of the patient’s lack of progress may not be immediately apparent [19]. A study of 100 trans-catheter aortic valve implantation (TAVI) patients, reported that anticipation for ECMO and institution of this mode of support prophylactically in the “high-risk” category on the EuroSCORE scale would potentially prevent the need for salvage VA ECMO, in a less optimal and controlled clinical setting, and carry better outcomes. In this study all-cause mortality occurred in none of the high-risk patients undergoing prophylactic VA ECMO (although *p* > 0.05) [37].

The literature demonstrates overwhelming evidence pointing towards reasonable survival rate to hospital discharge for patients undergoing ECMO for an otherwise universally fatal clinical condition. Furthermore reasonable intermediate-term and long-term survival rates as well as good quality of life have been reported in a few studies (Table 1) mainly for the survivors that do not manifest the APIs (see results). Major life threatening ECMO complications are common. We advocate a multidisciplinary team (MDT) approach to decision making for institution of ECMO in the context of refractory PCCS [29]. We recommend that given the ethical and cost implications, the surgeon, the anaesthetist, the on-call intensivist, the on-call perfusionist and a cardiac surgeon not involved in the operation (e.g. the on-call cardiac surgeon) should be involved in the decision making in whether or not to institute ECMO for refractory PCCS on a case-by-case basis [20]. We believe that due to the complexity of such cases protocols may not be an adequate substitute for an MDT approach to decision-making and management of such complex patients [5, 20]. Finally, in order to both preserve the patients’ autonomy and aid decision-making in the event of encountering refractory PCCS, we advocate that the possibility of the need for VA ECMO along with its pros and cons should be discussed with high-risk patients preoperatively and informed consent should be obtained in this regard.

## Conclusions

We believe that VA ECMO provides a survival benefit for a significant proportion of patients with refractory PCCS, which is invariably a fatal clinical state. For hospital survivors, a reasonable intermediate and long-term functional outcome can be expected albeit at the expense of prolonged and often ridden hospital stay. We identified advanced age, renal failure and prolonged VA ECMO support to be commonly reported APIs. This claim however could not be supported by meta-regression due to small patient numbers and heterogeneity of the patient populations. Risk stratification tools such as the EuroSCORE can be utilized to anticipate the need for prophylactic perioperative VA ECMO in the high-risk category. The reported APIs should be taken into consideration before institution of and ongoing treatment with this laborious, invasive and expensive therapeutic modality.

### Limitations

Most of the evidence available in the literature, including our analysis, pertains to older studies from the 1990s. Due to the nature of PCCS, randomization would not be appropriate and the studies typically constitute heterogeneous patient populations leading to skewing of the data. As for the pre-VA ECMO IABP application subgroup analysis, a few studies had not reported this data. In such studies we assumed that, in principal, all patients would have had IABP pre-VA ECMO institution. Hence the outcome of data analysis errs on the side of IABP usage. Small number of studies and heterogeneity of the patient populations meant that the statistical tests were not strong enough to detect statistical significance in our study. The data on EuroSCORE, rising lactate whilst on ECMO and the rate of obesity was not reported by enough studies to constitute a more objective analysis, hence deriving any solid conclusions regarding these indicators was difficult.

## Abbreviations

ACT: Activated clotting time
API: Adverse prognostic indicator
AS: Aortic stenosis
AVR: Aortic valve replacement
BiVAD: Biventricular assist device
CABG: Coronary artery bypass grafting
CHD: Congenital heart disease
CI: Cardiac index
CKD: Chronic kidney disease
CPB: Cardiopulmonary bypass
CVA: Cerebrovascular accident
ECMO: Extra-corporeal membrane oxygenator
GI: Gastrointestinal
IABP: Intra-aortic balloon pump
ICU: Intensive care unit
LMS: Left main stem coronary artery
LV: Left ventricular
LVAD: Left ventricular assist device
MCS: Mechanical circulatory support
MDT: Multidisciplinary team
MI: Myocardial infarction
MODS: Multi-organ dysfunction syndrome
MV: Mitral valve
MVR: Mitral valve replacement
MVR: Mitral valve replacement
NHS: National Health Service
NO: Nitric oxide
NYHA: New York Heart Association
OHT: Orthotopic heart transplantation
OHT: Orthotopic heart transplantation
PCCS: Postcardiotomy cardiogenic shock
PVD: Peripheral vascular disease
RV: Right ventricular
RVAD: Right ventricular assist device
SIADH: Syndrome of inappropriate anti-diuretic hormone
TAVI: Trans-catheter aortic valve implantation
TV: Tricuspid valve
TVR: Tricuspid valve replacement
UH: Unfractionated heparin
UK: United Kingdom of Great Britain
VA: Veno-arterial
VA: Veno-arterial
